# Necrological: Dr. Wagley, Dr. Paine, Dr. DeSteiger

**Published:** 1894-01

**Authors:** 


### NECROLOGICAL.

Death has been busy in the ranks of the medical profession in Texas recently, and the "insatiate archer" has picked off some of its brightest ornaments. Alas, those who devote their whole lives to battling with the fell destroyer must all, sooner or later, fall before his invincible power.

DR. C. F. PAINE, of Comanche, Texas, died at his home September 13, 1893. Dr. Paine was a conspicuous member of the Texas profession and of the State Medical Association. He attended nearly every meeting of the body, however remote from his home. For instance, when the meetings were held at Galveston, San Antonio or Houston, the entire length of the State, some seven hundred or more miles had to be twice traversed This fact alone testified the deep interest Dr. Paine felt in the progress of medical science. He was a son of Dr. F. T. Paine who was one of the pioneer physicians of Texas, and made a reputation in electro-therapeutics, being one of the first physicians in Texas to use electricity in the scientific treatment of disease.

Dr. C. F. Paine was a Mississippian by birth; born in De Soto county, in Mississippi, July 29, 1849; graduated in medicine in Tulane University, 1875; removed to Texas April 29, 1879, and located, residing there continuously, and actively engaged in practice till the time of his death. Dr. Paine was twice married; his first wife, to whom he was married in Arkansas, February 1, 1871, was Miss Mary Oliver; his second wife was Miss S. C. Redden, of Comanche county, Texas, to whom he was married September 26, 1883. He practiced medicine twenty-three years, nine years of which were spent in Mississippi.

Dr. Paine was a member of the Northwest Texas and the

Central Texas Medical Societies, as well as of the State Society. To each of these he contributed valuable papers.*

His death is a loss to the Texas profession and to his constituency. A genial gentleman and a skilled physician, every home in Comanche county will mourn his untimely death. Cut down in the prime or life his death was the greater shock, and saddens the hearts of a host of admiring friends.

DR. EDWARD DESTEIGER, of San Marcos, Tex., died December 10, after a protracted illness. Dr. DeSteiger was a graduate of Starling Medical College, Columbus. Ohio, of the class of 1876. He was the oldest resident physician of San Marcos. His son, Dr. John R. DeSteiger, a graduate of Hospital College Medicine (Med. Dep. Central University) Louisville, Ky., class of 1890, succeeds him in practice.

DR. JNO. L. WAGLEY, of Cleburne, Tex., a courtly gentleman and a most excellent man in every relation of life, a physician of high character and standing, met with a horrible death at Cleburne, on the afternoon of Dec. 13th. The following is the account given of the casualty in the press dispatches to the Austin *Statesman*. It was a great shock to the community and has created widespread grief and sorrow in the profession by whom the doctor was held in universal high regard and esteem. He was one of the staunchest workers in the State Medical Association and an unfaltering supporter of the Code and of the TEXAS MEDICAL JOURNAL:

"CLEBURNE, TEX., Dec. 13.—A most horrible accident happened here at 7:30 o'clock this evening when Dr. John Wagley, a well known physician, who has been practising medicine in Cleburne for thirty years, was run over by the north bound Santa Fe train. His head was severed from his body which was badly mangled all over. He had gone to the depot with his wife and son, who were going away. He attempted to cross the track in front of the train, which was moving out at the time, when the cowcatcher knocked him down and threw him on the track in front of the engine, which passed over his body. Besides Mrs. Wagley and her son, about one hundred people were on the depot platform and saw the accident. Several ladies fainted at the horrible sight. Dr. Wagley was the most prominent physicians in

---

[* The paper on Salix Niger, read before the State Medical Association at Houston, in 1885, and which Dr. Craig, in the Courier Record, attributes to Dr. C. F. Paine, was by the elder Dr. Paine (F. T).—ED.]

Cleburne and was well known throughout the State. He leaves a wife and a large family. One of his daughters was married last week and is in Galveston on a bridal tour."

